# Susceptible supply limits the role of climate in the early SARS-CoV-2 pandemic

**DOI:** 10.1126/science.abc2535

**Published:** 2020-05-18

**Authors:** Rachel E. Baker, Wenchang Yang, Gabriel A. Vecchi, C. Jessica E. Metcalf, Bryan T. Grenfell

**Affiliations:** 1 Princeton Environmental Institute, Princeton University, Princeton, NJ, USA.; 2 Department of Ecology and Evolutionary Biology, Princeton University, Princeton, NJ, USA.; 3 Department of Geosciences, Princeton University, Princeton, NJ, USA.; 4 Woodrow Wilson School of Public and International Affairs, Princeton University, Princeton, NJ, USA.; 5 Division of International Epidemiology and Population Studies, Fogarty International Center, National Institutes of Health, Bethesda, MD, USA.

## Abstract

Preliminary evidence suggests that climate may modulate the transmission of severe acute respiratory syndrome coronavirus 2 (SARS-CoV-2). Yet it remains unclear whether seasonal and geographic variations in climate can substantially alter the pandemic trajectory, given that high susceptibility is a core driver. Here, we use a climate-dependent epidemic model to simulate the SARS-CoV-2 pandemic by probing different scenarios based on known coronavirus biology. We find that although variations in weather may be important for endemic infections, during the pandemic stage of an emerging pathogen, the climate drives only modest changes to pandemic size. A preliminary analysis of nonpharmaceutical control measures indicates that they may moderate the pandemic-climate interaction through susceptible depletion. Our findings suggest that without effective control measures, strong outbreaks are likely in more humid climates and summer weather will not substantially limit pandemic growth.

## CORONAVIRUS

In some quarters, it is hoped that increased humidity and higher temperatures over the Northern Hemisphere in the summer will snuff out the 2020 severe acute respiratory syndrome coronavirus 2 (SARS-CoV-2) pandemic. In reality, the situation is likely to be more complicated than that. Baker *et al.* used a climate-dependent epidemic model to simulate the SARS-CoV-2 pandemic, testing different scenarios of climate dependence based on known coronavirus biology. Levels of susceptibility among the population remain the driving factor for the pandemic, and without effective control measures, the pandemic will persist in the coming months, causing severe outbreaks even in humid climates. Summer will not substantially limit pandemic growth.

*Science* this issue p. 315

## Teaser

A high proportion of susceptible people in a population is likely to outweigh any moderating effects of summer weather on virus transmission.

The severe acute respiratory syndrome coronavirus 2 (SARS-CoV-2) pandemic represents an unprecedented public health, social, and economic challenge. Sustained local transmission is present in multiple countries and in all continents, and the implications in terms of morbidity and mortality are expected to be severe (*1*, *2*). The role of seasonal and geographic climate variations in modulating the transmission of the virus has received increasing attention. Studies using a regression framework have found a role for temperature and relative and specific humidity in the transmission of SARS-CoV-2 (*3*–*7*), suggesting that cold, dry conditions increase the transmission of the virus. However, with limited data on the current epidemic, these early-stage results are inevitably inconclusive. Furthermore, the relative importance of climate drivers when compared with high population susceptibility during the pandemic stage of an emerging infection such as SARS-CoV-2 has not been fully characterized.

Climate affects the transmission of several directly transmitted pathogens (*8*). Specific humidity (the mass of water vapor in a unit mass of moist air) has been shown to be important for influenza transmission in both laboratory settings (*9*–*11*) and population-level studies (*12*). Respiratory syncytial virus (RSV), a childhood pathogen, has also been found to be dependent on specific humidity (*13*) and exhibits latitudinal correlations with climate (*14*). For both influenza and RSV, low specific humidity increases transmission, and epidemics tend to peak in the wintertime in northern latitudes. However, other directly transmitted infections exhibit different patterns (*15*), with enteroviruses, for instance, often peaking in the summer months (*16*).

Prior work on climate and directly transmitted diseases has typically considered endemic infections, such as seasonal influenza or RSV. Emerging pathogens, by contrast, have distinct dynamics driven by high population susceptibility (*17*). A key question is the extent to which seasonal and geographic climate variations are relevant in the pandemic phase of an emerging infection. Here, we build on known features of endemic human coronaviruses and other directly transmitted infections to probe this question. Although we do not yet know the climate sensitivity of SARS-CoV-2 transmission directly, data exists on four other coronaviruses that currently circulate in human populations. Two of these coronaviruses, human coronavirus HKU1 (HCoV-HKU1) and HCoV-OC43, are of the same betacoronavirus genus as SARS-CoV-2 (*18*).

We use data on HCoV-HKU1 and HCoV-OC43 from U.S. census regions to understand the potential climate dependence of betacoronavirus transmission (*19*). We fit a susceptible-infected-recovered-susceptible (SIRS) model to case data of HCoV-HKU1 and HCoV-OC43 where the fitted parameters include the climate dependence of transmission and the length of immunity after infection. All other parameters are fixed, based on values from Kissler *et al*. (*18*). Motivated by the climate dependence of influenza and RSV, we posit that transmission depends on specific humidity: We use population-weighted average climatology of specific humidity over 2014–2020 taken from the ERA5 reanalysis dataset (*20*), with population data from (*21*). We note that specific humidity is dependent on temperature through the Clausius-Clapeyron relation, and results using both variables have been found to be equivalent for other diseases (*13*). After fitting the model parameters, we run simulations of the SARS-CoV-2 pandemic under three scenarios. In the first scenario, we assume that SARS-CoV-2 has the same sensitivity to climate as influenza, based on a prior model from laboratory studies (*9*, *12*). In the second and third scenarios, we assume that SARS-CoV-2 has the same climate dependence and length of immunity as HCoV-OC43 and HCoV-HKU1, respectively. Although we assume that the climate dependence is the same as these three infections, our simulations use the basic reproduction number (*R*_0_) based on current estimates of SARS-CoV-2 (*18*, *22*).

**Fig. 1:**
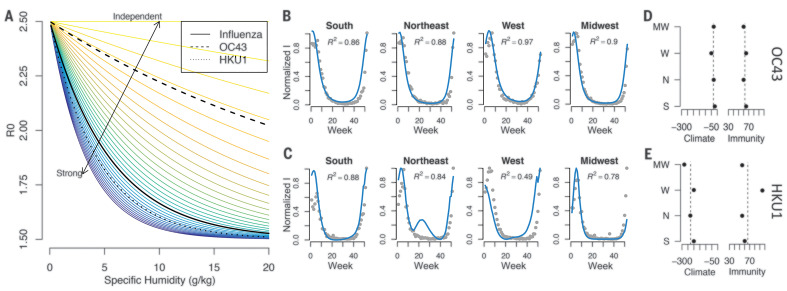
**Specific humidity and transmission.** (**A**) Colored lines represent different hypotheses for the relationship between climate and transmission for SARS-CoV-2. Values of *R*_0_ reflect SARS-CoV-2 estimates. The functional climate-dependence of influenza transmission, OC43 transmission, and HKU1 transmission is shown with solid, dashed, and dotted black lines, respectively. (**B** and **C**) A summary of seasonal model fits (blue line) for scaled average weekly cases (gray) of (B) OC43 and (C) HKU1. (Our model captures the biennial cycles of HKU1, shown in fig. S3, and detailed model fits for OC43, shown in fig. S2.) Coefficient of determination (*R*^2^) values are shown. I, number infected. (**D** and **E**) Fit results in terms of climate dependence and immunity length (weeks) for (D) OC43 and (E) HKU1, where mean fits are shown with dashed lines. MW, Midwest; W, West; N, North; S, South.

We first consider the seasonality of the endemic betacoronaviruses. Figure 1 shows the average seasonal pattern of endemic betacoronaviruses HCoV-OC43 and HCoV-HKU1 (hereafter OC43 and HKU1) for different regions in the United States. Cases of both diseases increase as specific humidity declines (fig. S1). We therefore assume that, to some extent, transmission will decline with specific humidity; however, the extent of the decline is yet to be determined. We characterize the link between specific humidity and the transmission of SARS-CoV-2 using plausible estimates derived from the two endemic betacoronaviruses as well as influenza. Figure 1A shows different potential functional forms for the climate-transmission relationship. Changes to specific humidity modulate *R*_0_ between a maximum wintertime value and a hypothesized lower bound, taken from prior studies (*18*, *22*). In the extreme cases, transmission (*R*_0_) either rapidly declines as specific humidity increases or has no relationship with specific humidity. The highlighted influenza relationship is based on laboratory studies using the guinea pig animal model (*9*–*11*) and later used to predict influenza epidemics in human populations (*12*). In this case *R*_0_ values correspond to SARS-CoV-2 estimates.

The other two scenarios in Fig. 1A correspond to the relationship between climate and OC43 and HKU1 transmission. We evaluate the functional form of this relationship by fitting our climate-driven SIRS model to U.S. case data for the two infections (Fig. 1, B and C, and figs. S2 and S3). Our results (Fig. 1, D and E) suggest a somewhat wide range of climate dependency for the two coronaviruses, with HKU1 having a much steeper response to specific humidity than OC43. Strong seasonal forcing has been linked to biennial outbreaks, as observed for HKU1 (fig. S3), in other respiratory pathogens (*13*) and implies endemic dynamics driven by herd immunity; however, this inference may be complicated by cross-protection from other circulating strains (*18*). Although there is some uncertainty in our estimates, simulating a pandemic outbreak using a range of climate-transmission dependencies allows us to explore a wide plausible range of potential climate effects.

**Fig. 2:**
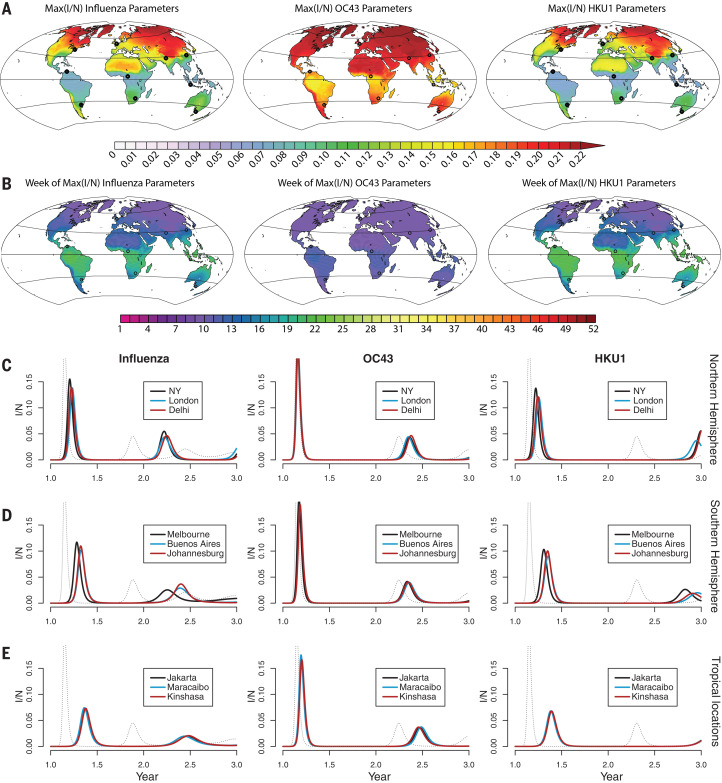
**Global model results and nine example trajectories.** (**A** and **B**) The (A) maximum number of infections per capita (I/N) and (B) timing of peak I/N for global locations. Black circles show locations where trajectories are explicitly shown. The color scale shows maximum I/N in (A) and week of peak I/N in (B). (**C** to **E**) Simulated pandemics are shown for cities in (C) the northern hemisphere, (D) the southern hemisphere, and (E) tropical locations. The dotted line represents a pandemic with no climate dependence. NY, New York.

We simulate a pandemic invasion for all locations (Fig. 2, A and B) and focus on the results for nine exemplar cities (Fig. 2, C to E), each with a very distinct mean and seasonal cycle of specific humidity (fig. S4). We stress that these initial simulations explore only the interaction of the epidemic (SIRS) model clockwork and seasonality in transmission; they do not address complexities of demography, control, and other environmental factors. In Fig. 2, C to E, we show the evolution of the simulated pandemic, holding population constant, for Northern Hemisphere, Southern Hemisphere, and tropical locations. The model assumes that the outbreak starts at the same time and that no control measures are in place, revealing only the effect of climate on pandemic size and duration. For the Northern Hemisphere locations, we do not see any substantial difference in pandemic size across all three scenarios, despite very different climates in New York, London, and Delhi. In the influenza and HKU1 scenarios, tropical locations experience a more sustained, lower-intensity pandemic than those in the Northern Hemisphere. These scenarios represent a stronger dependence on climate than the OC43 scenario, such that the lack of really dry conditions (low specific humidity) in tropical regions means that these locations do not experience the high transmission rates of the higher latitudes. However, the outbreak in the tropical cities remains substantial, and factors we do not explore here, such as population density, could further exacerbate the size of the epidemic.

We also simulate the pandemic in a range of Southern Hemisphere locations (Fig. 2D). We see only minor delays in the peak of Southern Hemisphere locations relative to those in the Northern Hemisphere (Fig. 2B), despite the 6-month shift in specific humidity seasonality between the two hemispheres (fig. S4). For the OC43 scenario, pandemics are temporally aligned across all locations and of similar magnitude. A stronger climate response for influenza and HKU1 parameters leads to slight regional differences. It is worth noting that our different scenarios also reflect a range of immunity lengths. The size of the pandemic peak is not affected by changes in immunity length (fig. S11), but the timing of later-stage outbreaks is partially dictated by this parameter. The differential timing of secondary peaks in the influenza and HKU1 scenarios, which have a similar climate dependence, is driven by this variability.

**Fig. 3:**
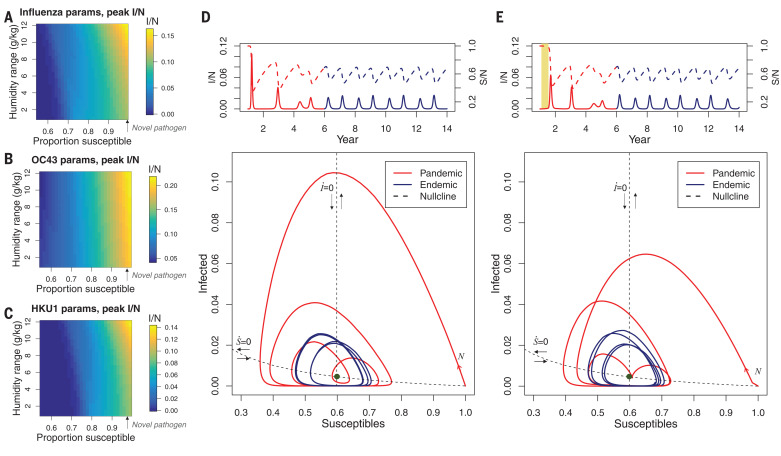
**Pandemic peak size depends on the proportion of the population that is susceptible.** (**A** to **C**) For the three scenarios, (A) influenza, (B) OC43, and (C) HKU1, the surface plot shows the dependence of maximum pandemic incidence per capita on the seasonal range of humidity and the proportion of the population that is susceptible, assuming mean humidity of New York. params, parameters. (**D**) The time series from pandemic to endemic outbreaks for an example location (Wuhan with HKU1 params) (top) and the equivalent SI phase plane of pandemic and epidemic trajectories (bottom). The two nullclines are from the unforced SIRS using mean *R*_0_. The green circle represents the equilibrium of the unforced model. S/N, proportion susceptible; $\dot{I}$, equilibrium infected; $\dot{S}$, equilibrium susceptible; *N*, total population. (**E**) The same trajectory but with a 6-month control period (reducing *R*_0_ to 1.1). The yellow shading indicates the timing of the control period.

During the pandemic stage of an emerging pathogen, the lack of population immunity—that is, high susceptibility—is a crucial driver. To illustrate this in the general case, we run our simulation model for different climates (represented by the seasonal range of humidity values a location experiences) and different levels of population susceptibility, using the mean specific humidity and seasonality of New York. Figure 3, A to C, shows the results in terms of the size of the pandemic peak. Although humidity range does modulate pandemic size, population susceptibility exhibits a much steeper gradient. For novel pathogens, such as SARS-CoV-2, the proportion of the population susceptible to infection may be close to 1. To illustrate the potential longer-term behavior of the pandemic, we plot a typical pandemic trajectory on the susceptible-infected (SI) phase plane (Fig. 3D). The initial pandemic trajectory (red) is relatively independent of seasonal forcing. This then gives way to the endemic attractor (blue), which oscillates around the equilibrium of the unforced model (green). These longer-term dynamics show a much stronger signature of seasonal forcing than the initial pandemic phase (figs. S9 and S10).

**Fig. 4:**
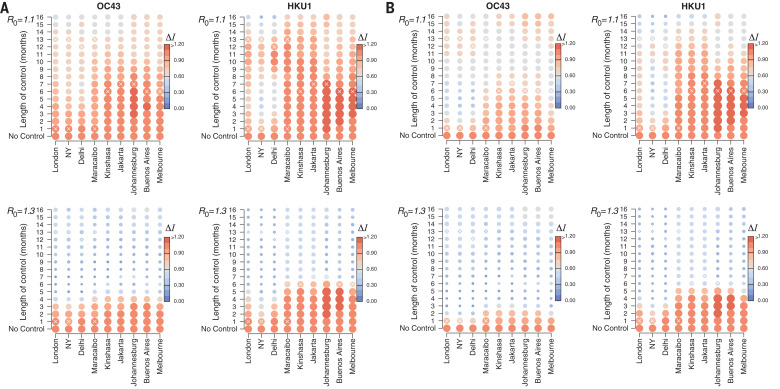
Interaction between control measures and the climate. (**A** and **B**) Four scenarios representing the interaction of different climate dependencies (OC43 and HKU1 params) with two potential control measures [*R*_0_ =1.1 and 1.3 in the control period, occurring (A) 1 month and (B) 6 weeks after pandemic start]. The size and color of the circles represent the size of peak incidence (within 2 years of pandemic start) relative to the no-control scenario. White crosses show the month of maximum climate-driven transmission, i.e., lowest specific humidity.

Figures 3E and 4 show a preliminary exploration of the impact of nonpharmaceutical control on the epidemic trajectory. In Fig. 3E, we show the SI phase plane where the HKU1 parameters of *R*_0_ are artificially controlled to *R*_0_ = 1.1 for a 6-month period. In this scenario, the control measures result in a moderate reduction in peak incidence as the outbreak is shifted to the summer months; however, high susceptibility still results in a substantial number of cases. In Fig. 4, we explore the interaction between the climate and control measures in more detail. We consider four scenarios: climate-dependencies based on OC43 and HKU1 as well as control measures where *R*_0_ = 1.1 or 1.3, representing limited transmission. For each scenario, we vary the length of the control measure and the location; however, for simplicity, we assume that all control measures start at two times: 4 and 6 weeks after the disease is introduced. We note that these control measures are simplified test cases and do not represent the local heterogeneity and efficacy of current controls, which are yet to be determined. These results show changes to peak incidence; changes to the number infected are shown in fig. S5.

For all control scenarios, we assume a degree of transmission during the control period, such that *R*_0_ > 1, resulting in an increase in population immunity over time. In the scenarios where* R*_0_ = 1.3, immunity builds faster and control measures work to reduce the pandemic peak after several months. In the *R*_0_ = 1.1 scenario, more time is required for population immunity to build such that the pandemic peak is reduced across all locations. In this scenario, as susceptibility declines, the climate plays a more substantial role in determining pandemic peak size (Fig. 4, A and B). When *R*_0_ = 1.1 in both the HKU1 and OC43 scenarios, releasing control measures close to the month of maximum transmission may result in a larger pandemic peak compared with the no-control scenario, particularly in the higher latitudes where transmission likely increases in the winter (fig. S6).

More broadly, our simulated control measures imply that the key determinant of reduced peak incidence is the extent to which population immunity builds over the control period, as demonstrated by the higher efficacy of the* R*_0_ = 1.3 control scenario in mitigating peak incidence. The climate plays a complex role in tuning the efficacy of potential control efforts, resulting in differential outcomes depending on location; however, population susceptibility remains a fundamental driver. Further exploration of these complexities may be necessary when evaluating location-specific controls. The timing of introduction and the efficacy of local control measures, as well as factors such as population density and contact patterns, could also shape future outcomes. Serological surveys, at the local level, will be important for tracking the build-up of immunity over time. Moreover, implementing control measures buys crucial time while vaccines and other treatments are developed.

There are several caveats to interpreting these results. Primarily, these simulations do not address location-specific factors such as spatial and social mixing patterns, contact networks, population density, and the specifics of control timing and efficacy. In particular, our results apply most closely to relatively well-mixed epidemics in large cities. Rural areas (with potentially lower *R*_0_; see figs. S7 and S8) may have a more delayed initial epidemic with complex consequences for ensuing interactions with climate drivers. Our model also does not account for potential cross-protection from other coronavirus infections (*18*). Cross-immunity may contribute to the seasonality of endemic coronaviruses, meaning that estimated climate drivers could be even weaker than we suggest and that our main findings are conservative (*16*, *23*). Finally, results from influenza and RSV suggest that high precipitation may play a role in driving transmission (*13*, *24*), particularly in tropical locations. Owing to limited data on betacoronaviruses from tropical locations, we have not been able to confirm whether a rainfall signal exists. Precipitation effects and other drivers such as schooling may also affect the epidemic trajectory, particularly after the pandemic. We further test for the sensitivity of our results to changes in core parameter values (materials and methods and figs. S7 to S11). These analyses suggest that our results are qualitatively robust to variations in climate dependency and weather fluctuations.

Our results suggest that although climate may play a role in modulating detailed aspects of the size and time scales of a pandemic outbreak within a particular location, population immunity is a much more fundamental driver of pandemic invasion dynamics. Although our HKU1 scenario presents a modest role for climate in terms of shifting the timing and intensity of the pandemic, a scenario with OC43 parameters is equally likely. In terms of the SARS-CoV-2 pandemic, our results imply that both tropical and temperate locations should prepare for severe outbreaks of the disease and that summertime temperatures will not effectively limit the spread of the infection. However, this does not mean that the climate is not important in the longer term. Endemic cycles of the disease will likely be tied to climate factors, and seasonal peaks may vary with latitude (figs. S3, S9, and S10). A more detailed understanding of climate drivers as well as immunity length will be crucial for understanding the implications of control measures. Furthermore, weather and near-term climate forecasts could be helpful for predicting secondary outbreaks after the initial pandemic phase has passed.

## Supplementary Materials

science.sciencemag.org/content/369/6501/315/suppl/DC1 Materials and Methods Figs. S1 to S11 References (*26*–*29*) MDAR Reproducibility Checklist

## References

[1] R. Verity, L. C. Okell, I. Dorigatti, P. Winskill, C. Whittaker, N. Imai, G. Cuomo-Dannenburg, H. Thompson, P. Walker, H. Fu, A. Dighe, J. Griffin, A. Cori, M. Baguelin, S. Bhatia, A. Boonyasiri, Z. M. Cucunuba, R. Fitzjohn, K. A. M. Gaythorpe, W. Green, A. Hamlet, W. Hinsley, D. Laydon, G. Nedjati-Gilani, S. Riley, S. van-Elsand, E. Volz, H. Wang, Y. Wang, X. Xi, C. Donnelly, A. Ghani, N. Ferguson, Estimates of the severity of COVID-19 disease. medRxiv 2020.03.09.20033357 [Preprint]. 13 March 2020; 10.1101/2020.03.09.20033357.

[2] R. Li, C. Rivers, Q. Tan, M. B. Murray, E. Toner, M. Lipsitch, The demand for inpatient and ICU beds for COVID-19 in the US: lessons from Chinese cities. medRxiv 2020.03.09.20033241 [Preprint]. 16 March 2020; 10.1101/2020.03.09.20033241.

[3] Q. Bukhari, Y. Jameel, Will coronavirus pandemic diminish by summer? SSRN 3556998 [Preprint]. 17 March 2020; 10.2139/ssrn.3556998.

[4] J. Wang, K. Tang, K. Feng, W. Lv, High temperature and high humidity reduce the transmission of COVID-19. SSRN 3551767 [Preprint]. 9 March 2020; 10.2139/ssrn.3551767.

[5] Sajadi M. M., Habibzadeh P., Vintzileos A., Shokouhi S., Miralles-Wilhelm F., Amoroso A., Temperature, humidity, and latitude analysis to estimate potential spread and seasonality of coronavirus disease 2019 (COVID-19). JAMA Netw. Open 3, e2011834 (2020). 10.1001/jamanetworkopen.2020.11834 32525550 PMC7290414

[6] W. Luo, M. S. Majumder, D. Liu, C. Poirier, K. D. Mandl, M. Lipsitch, M. Santillana, The role of absolute humidity on transmission rates of the COVID-19 outbreak. medRxiv 2020.02.12.20022467 [Preprint]. 17 February 2020; 10.1101/2020.02.12.20022467.

[7] Y. Ma, Y. Zhao, J. Liu, X. He, B. Wang, S. Fu, J. Yan, J. Niu, B. Luo, Effects of temperature variation and humidity on the mortality of COVID-19 in Wuhan. medRxiv 2020.03.15.20036426 [Preprint]. 18 March 2020; 10.1101/2020.03.15.20036426. PMC7142681 32408453

[8] Baker R. E., Mahmud A. S., Metcalf C. J. E., Dynamic response of airborne infections to climate change: Predictions for varicella. Clim. Change 148, 547–560 (2018). 10.1007/s10584-018-2204-4

[9] Shaman J., Kohn M., Absolute humidity modulates influenza survival, transmission, and seasonality. Proc. Natl. Acad. Sci. U.S.A. 106, 3243–3248 (2009). 10.1073/pnas.0806852106 19204283 PMC2651255

[10] Lowen A. C., Steel J., Roles of humidity and temperature in shaping influenza seasonality. J. Virol. 88, 7692–7695 (2014). 10.1128/JVI.03544-13 24789791 PMC4097773

[11] Lowen A. C., Mubareka S., Steel J., Palese P., Influenza virus transmission is dependent on relative humidity and temperature. PLOS Pathog. 3, 1470–1476 (2007). 10.1371/journal.ppat.0030151 17953482 PMC2034399

[12] Shaman J., Pitzer V. E., Viboud C., Grenfell B. T., Lipsitch M., Absolute humidity and the seasonal onset of influenza in the continental United States. PLOS Biol. 8, e1000316 (2010). 10.1371/journal.pbio.1000316 20186267 PMC2826374

[13] Baker R. E., Mahmud A. S., Wagner C. E., Yang W., Pitzer V. E., Viboud C., Vecchi G. A., Metcalf C. J. E., Grenfell B. T., Epidemic dynamics of respiratory syncytial virus in current and future climates. Nat. Commun. 10, 5512 (2019). 10.1038/s41467-019-13562-y 31797866 PMC6892805

[14] Pitzer V. E., Viboud C., Alonso W. J., Wilcox T., Metcalf C. J., Steiner C. A., Haynes A. K., Grenfell B. T., Environmental drivers of the spatiotemporal dynamics of respiratory syncytial virus in the United States. PLOS Pathog. 11, e1004591 (2015). 10.1371/journal.ppat.1004591 25569275 PMC4287610

[15] Martinez M. E., The calendar of epidemics: Seasonal cycles of infectious diseases. PLOS Pathog. 14, e1007327 (2018). 10.1371/journal.ppat.1007327 30408114 PMC6224126

[16] Takahashi S., Liao Q., Van Boeckel T. P., Xing W., Sun J., Hsiao V. Y., Metcalf C. J. E., Chang Z., Liu F., Zhang J., Wu J. T., Cowling B. J., Leung G. M., Farrar J. J., van Doorn H. R., Grenfell B. T., Yu H., Hand, foot, and mouth disease in China: Modeling epidemic dynamics of enterovirus serotypes and implications for vaccination. PLOS Med. 13, e1001958 (2016). 10.1371/journal.pmed.1001958 26882540 PMC4755668

[17] Gog J. R., Ballesteros S., Viboud C., Simonsen L., Bjornstad O. N., Shaman J., Chao D. L., Khan F., Grenfell B. T., Spatial transmission of 2009 pandemic influenza in the US. PLOS Comput. Biol. 10, e1003635 (2014). 10.1371/journal.pcbi.1003635 24921923 PMC4055284

[18] Kissler S. M., Tedijanto C., Goldstein E., Grad Y. H., Lipsitch M., Projecting the transmission dynamics of SARS-CoV-2 through the postpandemic period. Science eabb5793 (2020). 10.1126/science.abb5793 32291278 PMC7164482

[19] Centers for Disease Control and Prevention, Surveillance for common human coronaviruses (2020); www.cdc.gov/surveillance/nrevss/coronavirus/index.html.

[20] Hoffmann L., Günther G., Li D., Stein O., Wu X., Griessbach S., Heng Y., Konopka P., Müller R., Vogel B., Wright J. S., From ERA-Interim to ERA5: The considerable impact of ECMWF’s next-generation reanalysis on Lagrangian transport simulations. Atmos. Chem. Phys. 19, 3097–3124 (2019). 10.5194/acp-19-3097-2019

[21] NASA Socioeconomic Data and Applications Center (SEDAC), Gridded Population of the World, Version 4 (GPWv4): Population Density, Revision 11. Palisades, NY; https://sedac.ciesin.columbia.edu/data/set/gpw-v4-population-density-rev11 [accessed March 2020].

[22] S. W. Park, B. M. Bolker, D. Champredon, D. J. D. Earn, M. Li, J. S. Weitz, B. T. Grenfell, J. Dushoff, Reconciling early-outbreak estimates of the basic reproductive number and its uncertainty: framework and applications to the novel coronavirus (SARS-CoV-2) outbreak. medRxiv 2020.01.30.20019877 [Preprint]. 28 February 2020; 10.1101/2020.01.30.20019877. PMC7423425 32693748

[23] Kamo M., Sasaki A., The effect of cross-immunity and seasonal forcing in a multi-strain epidemic model. Physica D 165, 228–241 (2002). 10.1016/S0167-2789(02)00389-5

[24] Tamerius J. D., Shaman J., Alonso W. J., Bloom-Feshbach K., Uejio C. K., Comrie A., Viboud C., Environmental predictors of seasonal influenza epidemics across temperate and tropical climates. PLOS Pathog. 9, e1003194 (2013). 10.1371/journal.ppat.1003194 23505366 PMC3591336

[25] R. E. Baker, rebaker64/climate-cov: R1. Zenodo (2020); 10.5281/zenodo.3787766.

[26] Killerby M. E., Biggs H. M., Haynes A., Dahl R. M., Mustaquim D., Gerber S. I., Watson J. T., Human coronavirus circulation in the United States 2014–2017. J. Clin. Virol. 101, 52–56 (2018). 10.1016/j.jcv.2018.01.019 29427907 PMC7106380

[27] Gelaro R., McCarty W., Suárez M. J., Todling R., Molod A., Takacs L., Randles C., Darmenov A., Bosilovich M. G., Reichle R., Wargan K., Coy L., Cullather R., Draper C., Akella S., Buchard V., Conaty A., da Silva A., Gu W., Kim G.-K., Koster R., Lucchesi R., Merkova D., Nielsen J. E., Partyka G., Pawson S., Putman W., Rienecker M., Schubert S. D., Sienkiewicz M., Zhao B., The Modern-Era Retrospective Analysis for Research and Applications, Version 2 (MERRA-2). J. Clim. 30, 5419–5454 (2017). 10.1175/JCLI-D-16-0758.1 32020988 PMC6999672

[28] L. Bao, W. Deng, H. Gao, C. Xiao, J. Liu, J. Xue, Lv. Qi, J. Liu, P. Yu, Y. Xu, F. Qi, Y. Qu, F. Li, Z. Xiang, H. Yu, S. Gong, M. Liu, G. Wang, S. Wang, Z. Song, Y. Liu, W. Zhao, Y. Han, L. Zhao, X. Liu, Q. Wei, C. Qin, Lack of reinfection in rhesus macaques infected with SARS-CoV-2. bioRxiv 2020.03.13.990226 [Preprint]. 1 May 2020; . 10.1101/2020.03.13.990226

[29] Callow K. A., Parry H. F., Sergeant M., Tyrrell D. A., The time course of the immune response to experimental coronavirus infection of man. Epidemiol. Infect. 105, 435–446 (1990). 10.1017/S0950268800048019 2170159 PMC2271881

